# Relative validation of a food frequency questionnaire to estimate food intake in an adult population

**DOI:** 10.1080/16546628.2017.1305193

**Published:** 2017-03-29

**Authors:** Nina Steinemann, Leticia Grize, Katrin Ziesemer, Peter Kauf, Nicole Probst-Hensch, Christine Brombach

**Affiliations:** ^a^ Institute of Food and Beverage Innovation, Zurich University of Applied Sciences, Life Sciences and Facility Management, Waedenswil, Switzerland; ^b^ Epidemiology, Biostatistics and Prevention Institute, University of Zurich, Zurich, Switzerland; ^c^ Department of Epidemiology and Public Health, Swiss Tropical and Public Health Institute, Basel, Switzerland; ^d^ University of Basel, Basel, Switzerland; ^e^ Institute of Applied Simulation, Zurich University of Applied Sciences, Life Sciences and Facility Management, Waedenswil, Switzerland; ^f^ PrognosiX AG, Richterswil, Switzerland

**Keywords:** Food frequency questionnaire, weighed food record, validation study, dietary assessment, nutrient intake, food group intake, epidemiological studies

## Abstract

**Background**: Scientifically valid descriptions of dietary intake at population level are crucial for investigating diet effects on health and disease. Food frequency questionnaires (FFQs) are the most common dietary tools used in large epidemiological studies.

**Objective**: To examine the relative validity of a newly developed FFQ to be used as dietary assessment tool in epidemiological studies.

**Design**: Validity was evaluated by comparing the FFQ and a 4-day weighed food record (4-d FR) at nutrient and food group levels, Spearman’s correlations, Bland–Altman analysis and Wilcoxon rank sum tests were used. Fifty-six participants completed a paper format FFQ and a 4-d FR within 4 weeks.

**Results**: Corrected correlations between the two instruments ranged from 0.27 (carbohydrates) to 0.55 (protein), and at food group level from 0.09 (soup) to 0.92 (alcohol). Nine out of 25 food groups showed correlations > 0.5, indicating moderate validity. More than half the food groups were overestimated in the FFQ, especially vegetables (82.8%) and fruits (56.3%). Water, tea and coffee were underestimated (–14.0%).

**Conclusions**: The FFQ showed moderate relative validity for protein and the food groups fruits, egg, meat, sausage, nuts, salty snacks and beverages. This study supports the use of the FFQ as an acceptable tool for assessing nutrition as a health determinant in large epidemiological studies.

## Introduction

Dietary intake and, therefore, questions on dietary assessment for nutritional epidemiology play an important role in the worldwide discussion on chronic disease and general public health issues [1–5]. Among environmental and life-style determinants, nutritional behaviour represents a major target for the prevention of several non-communicable diseases, such as cancer, cardiovascular diseases, diabetes, chronic obstructive pulmonary disease and other chronic diseases [6–11]. A number of methods have been used to assess usual dietary intake at the population level [12]. However, the accuracy and reliability of measuring diet still presents an on-going challenge [12–14]. Although weighed food records and 24-hour recalls have been widely used, their substantial burden on respondents and their economic constraints make them inapplicable for most large epidemiological studies. Food frequency questionnaires (FFQs) are relatively inexpensive, put less burden on the respondents, and do not require trained interviewers [15,16]. Therefore, they represent the most commonly used tools in epidemiological studies [17]. However, due to lower accuracy, the information collected by FFQs needs to be compared with information collected by a more accurate dietary assessment method. This will be a measure of the relative validity of the FFQ in comparison with the reference method, i.e. to which degree the method captures what it is designed to measure [18]. Several approaches for the validation of FFQs exist. Because of their dissimilar error structures, weighed food records represent the gold standard as a reference method in FFQ validation studies [19].

A comparable online FFQ has been validated with a 4-day weighed food record (4-d FR) among adolescents, focusing on both the energy and macronutrient intake and validation at the food group level [20]. The results of this validation study showed good agreement for the energy and macronutrient intake except for protein, and a good agreement for frequently consumed foods at the food group level.

In the present study, we validated a FFQ in paper format to assess the dietary intake of adults versus a 4-d FR. In addition to the energy and macronutrients intake (carbohydrates, protein, fat and fibre), the food group intake was also examined.

The FFQ was designed to be implemented in a randomized Swiss population, the Swiss Cohort Study on Air Pollution and Lung and Heart Diseases in Adults (SAPALDIA). This population is diverse and consists of German-, French- and Italian-speaking participants, all representing different eating cultures. In order to depict eating patterns with one instrument (in all three national Swiss languages), we need a robust tool, which will be able to compile data in a valid and reproducible manner. In order to validate the tool, we chose an environment to mimic similar challenging circumstances to establish proof of the robustness and usability of the instrument. We therefore chose a German speaking, randomized sample which included all age groups representing the target population of the SAPALDIA cohort.

## Methods

### Study population and design

In October 2012, study participants were recruited through advertisements and via email, telephone and word of mouth in the area of Jena, Germany. Sixty subjects were enrolled in the validation study, taking place between November 2012 and January 2013. For inclusion in the study, subjects were required to be at least 18 years of age, without chronic diseases requiring medication and not pregnant or breastfeeding. Written informed consent was obtained from all subjects for participation in this validation study. Participants completed both a FFQ in paper format and a 4-d FR as the reference method, within a period of 4 weeks. Both methods will be described in detail below. The subjects participating in the study were not reimbursed apart from being allowed to keep the scales at the end of the assessment method. In addition, there was a raffle for eight vouchers each with a value of 25 euros.

### Dietary assessment

*The 4-d FR (reference method*): At the beginning of the study, participants filled in a 4-d FR. The 4 days had to consist of both weekdays and weekend days. The study population was randomized into two groups with 30 subjects who filled in the 4-d FR continuously from Wednesday to Saturday and the other 30 subjects from Sunday to Wednesday. A paper template was handed out to each participant, consisting of 8 pages: 2 pages for each day. Each sheet was sub-divided into four columns in which the food and beverages consumed were recorded as: amount in grams or millilitres, specified food, type of meal (breakfast, lunch, dinner, snacks) and general comments. The participants were asked to weigh each food item or meal prior to its consumption and to record the leftovers. They were instructed to use the scales for each meal, including out-of-the home consumption, i.e. restaurants and canteens (cafeterias). The participants returned the completed 4-d FR within a period of 1-3 weeks.

*Paper form FFQ (test method*): Subsequently, the 127-itemed, semi-quantitative paper form FFQ was handed out and filled in self-administered. The FFQ covered the period of the previous 4 weeks, and thus covered the time of the weighed food record. The FFQ was designed at the ZHAW (Zurich University of Applied Sciences) to assess the habitual food intake of adults and collected consumption information for 127 food items (www.ernaehrungserhebung.ch). The 127 food items were selected according to the most typically consumed food products in Switzerland and, in addition, complemented the findings of the MONICA study, the CoLaus study and household budget data [21–23]. The portion size of each food item was defined according to the data described in the MONICA study, including a standard portion size of ± 30% for a small and a big portion size, respectively, as in the National Nutritional Survey II in the Federal Republic of Germany [24,25]. Subjects were asked to indicate, on average, the frequency, portion size and number of portions of each food item (out of 127) they consumed during the previous 4 weeks. The frequency was asked in nine categories ranging from ‘never’ to ‘daily’. If a food item was eaten several times a day, participants were asked to take this into account indicating the number of portions. The participants indicated the portion size in the three categories ‘small’, ‘pre-set’ and ‘big’ (specified by pictures placed next to each food item to make the indication of portion sizes comparable among the participants). For each category, a metric amount in grams or decilitres/centilitres was assigned.

Additional information collected included preparation and cooking methods, use of specific types of oil, butter and margarine, and the take-out foods consumed. The FFQ also collected information on the frequency of use of dietary supplements. The FFQ was pretested on several user groups. In addition, a ‘users data sheet’ was handed out (together with the food record and the FFQ paper form) to collect demographic information (age, sex, height, weight, educational level, job position, residential area), as well as additional information on the current diet (e.g. weight reduction diet), physical activity, household size and smoking habits.

### Statistical analysis

#### Data pre-processing

Prior to data entry and food coding, the FFQ paper form and the 4-d FR were checked for completeness and possible errors. Two out of a total of 60 subjects did not return the questionnaires. Participants who completed fewer than 4 days of the 4-d FR were excluded, i.e. two out of the remaining 58 participants (completion rate = 3 days). After scanning the FFQ paper forms, each questionnaire was checked for completeness, missing values and structurally impossible answers (e.g. two boxes checked where only one should be checked). The following data management procedures included the sections on frequency, the number of portions and the portion size. If there were neither indications of frequency nor portion size nor number of portions, the frequency information ‘never’ was assigned to that food item. If at least one of frequency, portion size or number of portions was indicated, the following strategy was applied: if there were missing values of frequencies or number of portions, the mean value of the frequency or number of portions relating to that food item was entered. Missing values of portion sizes were corrected with an entry of a pre-set standard portion size. From a total of 58 questionnaires (58 × 127 × 3 = 22,098 possible entries), 43 (74%) FFQs showed missing information on the mentioned categories. However, in 32 of the 43 (74.4%) questionnaires there were fewer than five missing entries per questionnaire. The most frequent missing values were found in the number of portions (N = 93 over all questionnaires). Previous studies showed that there are in general fewer missing values in more frequently consumed foods [26].

To check for implausible energy intakes and to avoid a bias from wrongly reported food habits in the FFQ, the distribution of the total energy intake computed from the FFQ reports was examined. A cut-off was defined at the 75th percentile plus 1.5 times the interquartile range (3553.3 kcal) and the 25th percentile minus 1.5 times the interquartile range (190.9 kcal) [27]. This led to the exclusion of two FFQs with an over-reporting of energy intake (4250.3 kcal, 5414.3 kcal). The corresponding energy values in the 4-d FR for both excluded FFQs were well within the plausible range (2099.7 kcal, 2119.9 kcal).

#### Data post-processing

Based on the similarity of type of food and nutrient composition, the 127-food items listed in the FFQ were grouped into 25 predefined food groups, see the first column in Table 3.

The categorization corresponded to a similar grouping already used in the National Nutritional Survey II in the Federal Republic of Germany [25]. The mean intake of each food item per day was calculated using frequency, portion size and number of portions: Frequency × [number of portions × 100] × portion size /28. In order to receive the nutrient intakes per day, the calculated food data were linked to the Swiss Food Composition Database (www.naehrwertdaten.ch) and, where necessary, completed using the German Nutrient Data Base (www.bls.nvs2.de). The 4-d FR data was entered in an online input mask that was designed at the ZHAW (www.ernaehrungserhebung.ch). Therefore, each food item from the 4-d FR was matched to the corresponding FFQ food item.

#### Statistical methods

Correlation between macronutrients and food groups of 4-d FR and FFQ were assessed with Spearman’s rho, since some of the macronutrients showed clear deviations from normally distributed residuals to a linear model (assessed through the Shapiro–Wilk test, Kolmogorov–Smirnoff test and QQplot).

Descriptive statistics for energy, nutrients and food groups intake are presented as means, medians and interquartile ranges. To evaluate the agreement between the FFQ and 4-d FR, the mean difference and percentage difference were calculated as the mean of all individual differences between the FFQ and 4-d FR ([Mean (FFQ – 4-d FR)]/[mean (4-d FR)].

For the examination of relative validity, the Spearman’s correlations were corrected for the day-to-day variation within-person using the de-attenuation method [19]. The corrected correlation, *r_c_*, was calculated using the following formula:

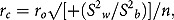

where r*_o_* is the observed correlation, *S^2^_w_*/*S^2^_b_* is the ratio of the within- and between-person variances and *n* is the number of replicates per person for the given variable. Within-person variation and between person variation were calculated from replicated 4-d FR.

For visualization, Bland–Altman diagrams and Box–Whisker plot were drawn. The Wilcoxon rank-sum test was used to examine reporting behaviour between participants groups. The statistical analysis was calculated using R version 3.0.1, SAS version 9.4 (2012–2012 SAS Institute Inc., Cary NC, USA) and Microsoft® Excel 2007. P values less than 0.05 were considered significant, all tests were performed two sided.

## Results

The characteristics of the 56 study participants are given in Table 1. The mean age was 40 years, ranging from 22 to 85 years and 60.7% were women. The mean height was 172.5 cm and the mean weight 72.3 kg. The mean body mass index was 24.2, ranging from 19.8 to 32.0.

**Table 1.** Characteristics of participants of the validation study by gender.

Characteristic*	Male	Female	Total
*n (%)*	22 (39.3)	34 (60.7)	56
Age, *years*	40.9 ± 19.7	39.9 ± 18.1	40.0 ± 18.6
Weight, *kg*	81.0 ± 9.7	69.6 ± 19.0	72.3 ± 9.1
Height, *cm*	181.4 ± 4.7	166.7 ± 5.6	172.5 ± 5.2
Body mass index, *kg/m^2^*	24.6 ± 2.8	25.1 ± 7.1	24.2 ± 3.0
PAL^†^	1.8 ± 0.2	1.7 ± 0.2	1.8 ± 0.2
Smoking, ever, *n (%)*	7 (31.8)	6 (17.6)	13 (23.2)
Highest level of education completed, *n (%)*			
Compulsory education	0 (0.0)	1 (2.9)	1 (1.8)
Secondary school	3 (13.6)	11 (32.4)	14 (25.0)
Tertiary degree	19 (86.4)	22 (64.7)	41 (73.2)
Place of residence, *n (%)*			
Urban	20 (90.9)	25 (73.5)	45 (80.4)
Rural	2 (9.1)	9 (26.5)	11 (19.6)

PAL, Physical Activity Level.

*Values are expressed as mean ± standard deviation or n (%)

^†^Expressed as a multiple of 24-hour basal metabolic rate [28]

The energy and macronutrient intake as reported in the FFQ was compared to that of the 4-d FR. Table 2 shows the means, medians and interquartile ranges for both instruments. Their mean and percentage differences are also given, as well as the correlations (Spearman’s rho) between the two methods, including the variance ratio and the de-attenuated (corrected) correlation coefficients. The final analysis included 54 subjects. The mean differences between FFQ and 4-d FR for carbohydrates, fibre and protein intake were positive, and negative for energy and fat intake. The correlations of intake derived from FFQ versus 4-d FR ranged between 0.27 (for carbohydrates) and 0.55 (for protein). Except for carbohydrates, all correlations were statistically significant.

**Table 2.** Relative validity of daily energy and macronutrient intakes estimated by the FFQ and 4-d FR, and correlations between FFQ and 4-d FR (n = 54) .

Macronutrient	FFQ intake			4-d FR intake			Mean difference (FFQ – 4-d FR)		Correlation between methods		
	Mean	Median	IQR†	Mean	Median	IQR†	Mean	*%‡*	Spearman’s r	Variance ratio§	Corrected Spearman’s r
Energy (kcal/d)	1858.7	1821.5	1432.0–2262.4	1908.8	1778.7	1543.6–2058.5	−50.2	−2.6	0.32*	0.97	0.36
Carbohydrates (g/d)	250.2	240.1	184.1–302.8	183.6	184.1	150.6–201.2	66.6	36.3	0.24	1.20	0.27
Fibre (g/d)	26.2	23.9	19.5–30.6	21.1	18.4	15.0–23.4	5.1	24.3	0.41**	0.64	0.44
Protein (g/d)	89.8	94.3	68.8–110.9	73.1	71.6	59.4–91.0	16.8	23.0	0.46***	1.79	0.55
Fat (g/d)	67.3	66.6	48.7–78.6	77.3	72.6	58.0–96.3	−10.0	−12.9	0.37**	1.26	0.42

*P < 0.05, **P < 0.01, ***P < 0.001

†IQR = Interquartile range = 25th percentile to 75th percentile

‡{[Mean (FFQ – 4-d FR)]/[mean (4-d FR)]} x 100

§Variance within subjects/Variance between subjects

Spearman correlation coefficient was adjusted using the de-attenuation method [19]

The ratio of within- and between-person variance calculated from the 4-d FR was between 0.64 and 1.79, and the de-attenuated (corrected) correlation coefficients were similar or slightly higher than the crude correlations (Table 2).

**Figure 1.**
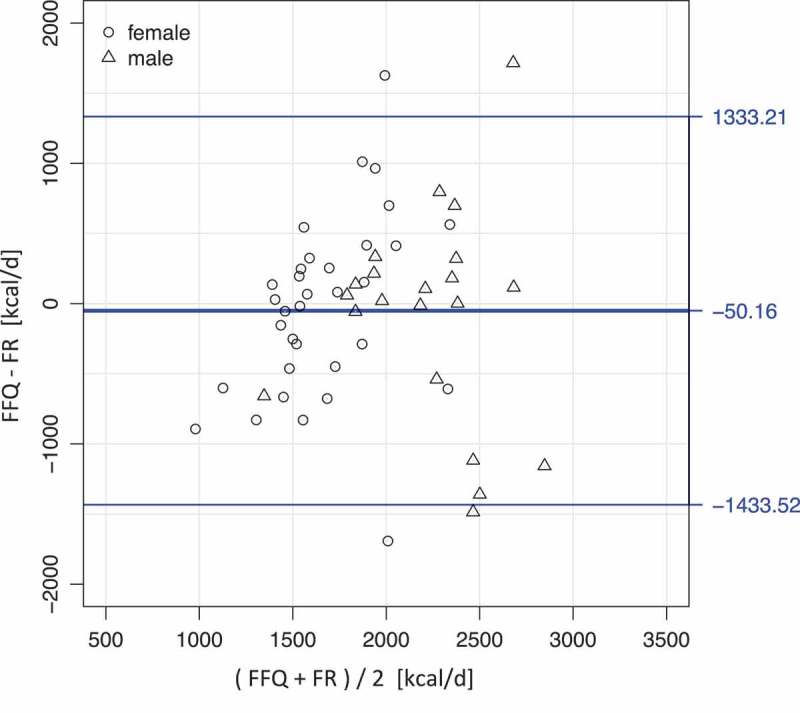
Bland–Altman plot of the energy intake as computed from 4-day weighed food record (4-d FR) and food frequency questionnaire (FFQ) reports. (Calculated for the whole sample, but different symbols label values for male and female participants.)

To examine the agreement in energy intake between the 4-d FR and FFQ, a Bland-Altman plot is presented in Figure 1. On average, the energy intake in the FFQ was slightly lower (50.2 kcal) than reported in the 4-d FR. A slight tendency for larger (absolute) differences between the instruments with increasing energy intake was observed for both men and women. Reporting behaviour between men and women did not differ (*P *= 0.90, Wilcoxon rank sum test), even though male participants reported higher energy intakes with both instruments (*P *< 0.0001, Wilcoxon rank sum test).

Table 3 shows the comparison of the food group intake as reported in the FFQ and 4-d FR, overall and stratified by gender, sorted by the magnitude of Spearman’s rho.

**Table 3.** Relative validity of food groups intake (g/d) estimated by the FFQ and 4-d FR (overall and by gender), and correlations between FFQ and 4-d FR (n = 54). The ordering is according to decreasing overall Spearman’s rho.

Food Groups	FFQ intake			4-d FR intake			Mean difference (FFQ – 4-d FR)		Correlation between methods		
	Mean	Median	IQR†	Mean	Median	IQR†	Mean	%‡	Spearman’s r	Variance ratio§	Corrected Spearman’s r
Alcoholic Beverages	111.6	48.2	14.3–161.4	127.9	0.0	0–250	−16.3	−12.7	0.71***	2.70	0.92
*Men*	206.1	150.7	65.7–260.0	227.9	125.0	0–387.5	−21.8	−9.6	0.68**	3.43	0.93
*Women*	51.5	35.7	7.1–64.3	64.2	0.0	0–100	−12.8	−19.9	0.65***	2.17	0.81
Meat	96.4	84.4	42.9–152.1	73.1	57.4	34.0–111.8	23.4	32.0	0.68***	–	–
*Men*	107.3	87.9	59.3–154.3	96.4	100.5	57.5–133.3	10.9	11.3	0.49*	–	–
*Women*	89.6	69.7	32.1–122.1	58.3	37.8	21.3–77.5	31.3	53.7	0.69***	–	–
Water, tea and coffee	1228.9	1075.0	725–1600	1429.3	1285.1	900.0–1794.8	−200.4	−14.0	0.66***	0.29	0.68
*Men*	1043.9	857.1	616.07–1325	1305.9	1182.0	838.3–1522.0	−262.0	−20.1	0.84***	0.39	0.88
*Women*	1346.7	1250.0	771.43–1650	1507.8	1320.3	915.0–2177.5	−161.2	−10.7	0.55**	0.25	0.57
Soft drinks with sugar	94.7	42.9	21.4–107.1	153.0	22.8	0–225.0	−58.3	−38.1	0.66***	1.80	0.79
*Men*	122.3	50.0	21.4–142.9	179.8	100.0	0–360.8	−57.5	−32.0	0.73***	1.96	0.89
*Women*	77.2	42.9	14.3–85.7	136.0	0.0	0–198.3	−58.8	−43.3	0.59***	1.66	0.70
Fruits	284.1	191.3	114.3–371.6	181.7	123.8	55.0–273.5	102.4	56.3	0.59***	0.66	0.64
*Men*	265.5	180.0	110.7–297.9	201.5	119.3	59.5–273.5	64.0	31.8	0.57**	0.40	0.60
*Women*	295.8	201.8	117.1–379.3	169.1	162.0	55.0–231.3	126.8	75.0	0.58***	1.15	0.66
Sausage	35.2	25.9	12.5–44.7	31.6	32.5	5.8–41.3	3.6	11.4	0.59***	1.00	0.66
*Men*	49.9	38.6	25.7–65.4	40.8	38.5	24.5–51.8	9.1	22.3	0.50*	1.19	0.57
*Women*	25.9	17.1	9.6–34.6	25.8	26.3	4.5–37.8	0.1	0.4	0.44*	2.62	0.57
Sweet spreads	21.1	12.5	4.5–30.4	13.0	7.9	0–24.0	8.1	62.6	0.49***	–	–
*Men*	23.8	21.4	5.4–31.3	15.9	14.5	0–25.8	7.9	50.0	0.33	1.87	0.40
*Women*	19.4	12.5	4.5–26.8	11.1	5.5	0–14.0	8.2	74.1	0.58***	–	–
Soft drinks without sugar	23.0	0.0	0.0	38.0	0.0	0.0	−15.0	−39.5	0.48***	0.27	0.50
*Men*	5.8	0.0	0.0	0.0	0.0	0.0	5.8	–	–	–	–
*Women*	34.0	0.0	0.0	62.2	0.0	0.0	−28.2	−45.4	0.55**	0.28	0.57
Cereals and grains	42.9	30.3	21.4–55.7	83.3	51.5	0–150.0	−40.4	−48.5	0.42**	1.69	0.50
*Men*	54.4	51.4	26.8–77.2	72.0	37.5	0–110.0	−17.6	−24.4	0.56**	1.40	0.65
*Women*	35.6	28.9	20.4–48.2	90.5	55.0	0–150.0	−54.9	−60.6	0.40*	2.35	0.50
Egg	21.6	15.5	11.8–35.4	24.2	15.0	0–35.0	−2.5	−10.5	0.41**	2.37	0.52
*Men*	26.1	23.6	11.8–35.4	40.3	32.3	0–54.8	−14.2	−35.3	0.56**	2.39	0.71
*Women*	18.8	11.8	5.9–23.6	13.9	13.0	0–18.8	4.9	35.1	0.26	6.67	0.42
Bread	145.5	137.5	92.9–182.1	134.9	133.4	96.3–173.8	10.6	7.9	0.40**	1.70	0.48
*Men*	162.3	140.0	100.0–214.3	160.6	163.0	132.5–192.0	1.7	1.1	0.14	1.22	0.16
*Women*	134.9	125.9	80.4–158.2	118.6	103.3	91.0–146.5	16.3	13.7	0.55**	2.68	0.71
Meat alternatives	2.2	0.0	0.0	2.8	0.0	0.0	−0.6	−21.4	0.40**	2.35	0.50
*Men*	2.9	0.0	0.0	6.1	0.0	0.0	−3.2	−53.0	0.35	2.02	0.43
*Women*	1.8	0.0	0.0	0.8	0.0	0.0	1.1	140.0	0.47**	–	–
Dairy products	152.0	124.4	67.9–177.7	144.4	117.0	50.0–199.5	7.6	5.2	0.40**	0.32	0.42
*Men*	187.4	150.0	67.9–225.9	167.0	150.0	93.8–233.8	20.4	12.2	0.42	1.08	0.47
*Women*	129.4	108.9	74.3–157.1	130.0	96.8	28.0–187.5	−0.6	−0.4	0.32	0.20	0.33
Nuts	4.3	1.3	0–6.4	2.5	0.0	0.0	1.8	73.0	0.37**	5.98	0.58
*Men*	4.6	2.1	1.1–6.4	2.0	0.0	0.0	2.7	136.5	0.18	0.72	0.20
*Women*	4.1	1.1	0–6.4	2.8	0.0	0.0	1.3	45.1	0.46**	–	–
Salty snacks	1.8	0.0	0–2.1	3.7	0.0	0.0	−1.9	−50.5	0.32*	7.12	0.53
*Men*	2.7	1.1	0–3.2	3.5	0.0	0.0	−0.8	−23.5	0.45*	2.52	0.57
*Women*	1.3	0.0	0–1.6	3.8	0.0	0.0	−2.5	−66.4	0.28	–	–
Fish	24.4	20.4	8.2–34.3	14.5	0.0	0–20.0	9.9	68.1	0.31*	–	–
*Men*	30.8	23.6	12.9–37.5	11.3	0.0	0–9.0	19.4	171.2	0.37	–	–
*Women*	20.3	19.3	6.4–27.9	16.5	13.5	0–22.5	3.8	23.0	0.34	–	–
Potato	63.6	51.7	28.9–90.0	92.4	69.8	0–135.0	−28.8	−31.2	0.31*	5.36	0.47
*Men*	82.7	78.2	48.2–106.1	116.7	132.0	0–182.0	−34.0	−29.1	0.32	4.40	0.46
*Women*	51.4	43.4	24.7–67.5	77.0	61.5	19.5–121.3	−25.5	−33.2	0.31	7.68	0.53
Vegetables	305.0	271.6	170.9–426.4	166.9	137.3	48.3–243.3	138.1	82.8	0.29*	1.84	0.35
*Men*	333.8	267.9	188.6–492.9	190.8	164.5	48.3–294.0	143.0	75.0	0.30	1.40	0.35
*Women*	286.6	275.3	170.9–378.2	151.7	136.3	62.3–200.0	135.0	89.0	0.25	2.56	0.32
Dessert	60.2	48.5	28.3–78.1	56.2	50.0	16.8–81.0	4.0	7.1	0.25	2.66	0.32
*Men*	71.1	48.1	37.5–84.8	53.9	44.0	18.8–76.0	17.2	32.0	−0.04	2.11	−0.05
*Women*	53.2	52.4	21.3–72.8	57.7	56.4	16.8–92.3	−4.5	−7.8	0.43*	3.20	0.58
Cheese	57.7	44.2	33.0–61.1	38.1	28.5	16.5–54.0	19.7	51.6	0.25	3.85	0.35
*Men*	62.7	38.2	34.3–54.1	45.5	35.2	21.5–67.5	17.2	37.9	0.33	5.16	0.50
*Women*	54.6	49.3	33.0–66.4	33.4	23.5	16.5–47.3	21.2	63.5	0.21	3.38	0.29
Preparation fats and savoury spreads	15.9	10.9	5.9–23.6	24.4	19.9	7.3–34.3	−8.5	−34.7	0.23	0.62	0.25
*Men*	18.8	12.9	7.5–25.0	27.9	26.0	10.3–36.5	−9.1	−32.5	0.17	0.56	0.18
*Women*	14.1	8.9	5.9–18.9	22.2	13.3	6.0–27.5	−8.1	−36.5	0.22	0.65	0.24
Composite foods	45.7	21.4	2.9–57.9	29.8	0.0	0.0	15.9	53.5	0.22	5.23	0.33
*Men*	69.0	45.4	5.7–122.2	42.7	0.0	0–85.0	26.3	61.6	0.39	11.54	0.77
*Women*	30.9	15.5	2.9–40.0	21.6	0.0	0.0	9.3	43.2	0.05	3.08	0.07
Sauce	12.2	9.3	4.9–15.4	36.4	26.9	0–55.8	−24.2	−66.4	0.15	9.49	0.28
*Men*	10.3	9.4	5.7–12.9	40.8	32.5	0–72.5	−30.5	−74.7	0.17	7.15	0.28
*Women*	13.4	9.1	4.9–20.0	33.6	23.8	0–50.0	−20.1	−60.0	0.17	10.90	0.33
Legumes	4.8	2.1	0–6.4	2.0	0.0	0.0	2.8	139.6	0.13	1.84	0.16
*Men*	5.3	3.2	0–6.4	3.0	0.0	0.0	2.3	78.0	0.13	1.40	0.15
*Women*	4.5	2.1	0–4.3	1.4	0.0	0.0	3.1	223.1	0.16	2.56	0.20
Soup	18.1	8.9	0–21.4	29.8	0.0	0.0	−11.6	−39.1	0.06	4.41	0.09
*Men*	12.8	7.1	0–21.4	16.0	0.0	0.0	−3.2	−20.2	0.01	4.38	0.01
*Women*	21.5	10.7	3.6–21.4	38.5	0.0	0.0	−17.0	−44.1	0.06	4.25	0.09

*P < 0.05, **P < 0.01, ***P < 0.001

†IQR = Interquartile range = 25th percentile to 75th percentile

‡{[Mean (FFQ – 4-d FR)]/[mean (4-d FR)]} x 100

§Variance within subjects/Variance between subjects. The ratio is not given when the variance between subjects is zero.

The Spearman correlation coefficient was adjusted using the de-attenuation method [19]

The corrected Spearman correlation coefficients ranged from 0.92 (alcohol) to 0.09 (soup). All correlations were significant except those for dessert, cheese, preparation fats and savoury spreads, composite foods, sauces, legumes and soups. Those food groups with a lower or non-significant correlation tended to include less frequently consumed foods, e.g. legumes and sauces. The correlations of 18 (72%) out of a total of 25 food groups were significant.

The mean difference between FFQ and 4-d FR varied among intakes, and there were almost as many foods that were underestimated (n = 12) as overestimated (n = 13) when compared with the reference method (Table 3). In general, frequently consumed foods such as bread, meat, fruits, vegetables, dairy products, cheese and sweet spreads were overestimated in the FFQ in comparison to the intakes assessed by the 4-d FR. No gender differences were observed for these food groups except for dairy products and dessert, which showed an underestimation in the FFQ for women compared to men (−0.6 g v. 20.4 g, −4.5 g v. 17.2 g). Vegetable and fruit intake were particularly overestimated by the FFQ by 138.1 g (82.8%) and 102.4 g (56.3%), respectively.

Food intakes that were underestimated in the FFQ comprised beverages (water, tea and coffee, soft drinks with and without sugar, alcoholic beverages), soup, sauce, preparation fats and savoury spreads, salty snacks, meat alternatives, eggs and cereals and grains. The lowest degree of underestimation was observed for water, tea and coffee with −200.4 ml (−14.0%).

Regarding gender, differences were found only for meat alternatives and soft drinks without sugar. Women, on average underestimated their intake of soft drinks without sugar (−28.2 g v. 5.8 g in men), while men on average underestimated their consumption of meat alternatives in the FFQ (−3.2 g v. 1.1 g in women).

**Figure 2.**
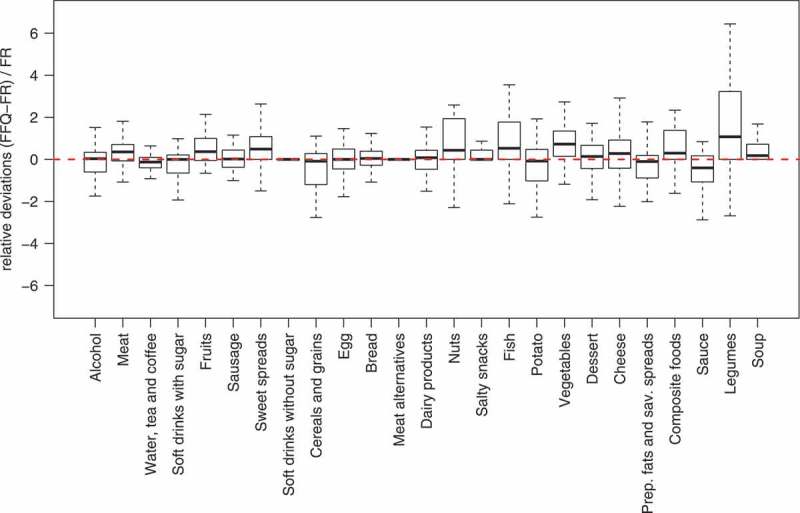
Participants’ relative differences in the food frequency questionnaire (FFQ) and the 4-day weighed food record (4-d FR) for each food group. The dashed line gives the zero difference between the medians of the two instruments. The unsystematic reporting difference between the two instruments is shown as the spread of the distributions indicated by the width of the boxes and the range of the whiskers.

In addition, the relative deviations of FFQ and 4-d FR are shown for each food group (Figure 2). In order to obtain comparability among the food groups, differences between FFQ and 4-d FR were divided by the mean reported intake value of the corresponding food group in the 4-d FR. The ordering of the food groups on the x-axis is according to decreasing magnitudes of Spearman’s rho (see Table 3).

## Discussion

This study focused on assessing relative validity of a paper form FFQ with a 4-d FR. The validity was assessed both, at the macronutrient and food group levels. From the 60 eligible participants, 58 completed the 4-d FR and FFQ according to the experimental design, but 56 subjects were considered for the analysis.

The relative validity of the FFQ, compared to the 4-d FR, varied among intakes of energy, macronutrients and food groups (Tables 2 and 3). The FFQ overestimated as well as underestimated the absolute intake of various nutrients and foods, which was comparable to other validation studies [29,30]. We observed that in general, frequently consumed foods tended to be overestimated in the FFQ compared to the 4-d FR, in particular vegetables and fruit intake, as reported in other FFQ validation studies [13,31]. Food items consumed daily (e.g. bread, dairy products) are better estimated by the FFQ as described in other studies [32,33]. These food groups may represent in general more frequently consumed foods for this study population, as they reflect common dietary habits. In contrast, food groups such as soup, sauce, preparation fats and savoury spreads and meat alternatives were underestimated in the FFQ when compared with the 4-d FR. These items may include rather rarely consumed foods, on the other hand, they may include food groups that are difficult to estimate portion size and rather tended to be ignored (e.g. sauce and preparation fats and savoury spreads). Furthermore, it should be considered that information on some of the food items was collected in a predefined manner in the FFQ compared to the open-end tool of the 4-d FR, where food items were weighed right at the time of consumption. For example, preparation fats and savoury spreads may have not been reported in the FFQ.

The application of correlation coefficients to assess relative validity in FFQ validation studies is still under debate, but there is a common agreement that correlations above 0.5 are moderate or good, and that correlations below 0.4 indicate a low degree of linear correlation [18,34]. Therefore, nine out of 25 food group intakes can be considered to have an acceptable validity for assessing intakes on a group level (all statistically significant, Table 3).

The correlation coefficients for energy and macronutrient intakes showed in general similar or lower values than those observed in other studies [9,13,29,31]. Protein and fibre intakes exhibited good correlations with values of 0.55 and 0.44. The lowest degree of linear association was found for carbohydrates (r = 0.27), which was also considerable at the food group level for legumes (r = 0.16), vegetables (r = 0.35) and desserts (r = 0.32). This finding may be related to the fact that some of the foods contributing to carbohydrate intake are consumed less frequently than weekly or only by a limited number of persons. Similarly, only five persons reported the consumption of legumes in the 4-d FR. Several persons reported legumes intake only once a month in the FFQ. The FFQ retrospectively assesses the diet covering the previous 4 weeks and the 4-d FR prospectively covers the actual dietary intake of 4 consecutive days.

Results obtained through the Bland–Altman method for energy intake showed slightly lower intakes on average for the FFQ than reported in the 4-d FR (50.2 kcal), with a slight tendency for larger (absolute) differences between the instruments with increasing energy intakes. This result could be partly explained by a higher tendency of underreporting in the FFQ for calorie-dense foods compared with the 4-d FR. Similar findings were reported in another study [33].

The results of this study point to relevant differences in reporting food intake between men and women. Compared to men, women reported a significantly higher intake of meat, fruits, sweet spreads and cheese in the FFQ compared with the 4-d FR. In response to social desirability, it is well known that women may be more likely to over-report food items related to a positive health image, e.g. fruits and vegetables, whereas sweets and cakes are usually associated with a rather negative health image and thus tend to be underreported [35]. In addition, the FFQ used for this study included a list of several fruits (n = 15) that also could lead to an over-reporting of fruit intake, as discussed elsewhere [36]. This poses a challenge to participants in estimating the overall fruit consumption [36]. Similar findings were observed for meat (n = 8) and cheese (n = 7) in this study. Additionally, the order of requested food items in the FFQ (e.g. meat is asked at the first position) could explain the significant differences between the two instruments.

For cereals and grains, women reported a significantly lower intake in the FFQ than in the 4-d FR, compared to men. Irrespective of the gender difference, reporting the portion sizes of these food items in the FFQ (e.g. noodles, rice, corn) could have been a challenge due to difficulties in the volume estimation by means of the food pictures.

There are some limitations in our study. First, the study participants from Jena, Germany may not be representative of the target Swiss population, for which the FFQ was designed. Therefore, this fact has to be kept in mind when citing this validation.

As previously discussed, the applied assessment tools contain several limitations. Despite the fact that the weighed food record (FR) is often denoted as the gold standard, it might cause a bias that has to be considered. On the one hand it is an invasive instrument that can induce changes in dietary habits, on the other hand it may not capture longer-term dietary patterns well. The FFQ in contrast, even though aiming at capturing food intake over longer time periods, faces the challenge of recall and difficulties in estimating portion size [19].

Due to the short sequence of data collection between the 4-d FR and FFQ, the awareness of an individual’s food habits could potentially affect the way the FFQ is filled in and therefore might also result in inflated correlations. A solution to this problem could be to let half of the group fill out the FFQ first and the other half to fill out the 4-d FR first.

Both instruments are time consuming for participants. While the FFQ is only filled in once and takes about 30–45 minutes, the time investment related to the FR is higher. It is an open-end tool performed several times per day for a fixed period of time and thereby puts a higher burden on daily life for weighing and recording food intake. In order to minimize the respondents’ burden, the use of emerging technologies, e.g. internet – based assessment tools presents a promising approach to tackle this challenge [37].

As already mentioned, an additional limitation of the FFQ could be the large number of listed food items within the food groups (from 1 for legumes to 22 for vegetables). This leads to a high variety of level of detail in the different food groups. Food groups including more items may lead to a cumulative effect and a tendency for over-reporting regarding that specific food group (e.g. fruits). Conversely, food groups containing only one item (e.g. egg) may lead to an underreporting effect due to the aggregation of foods (e.g. scrambled egg, fried egg, etc.) to the main group. This presents a challenge in the estimation of food intake.

In addition, the seasonality aspect must be taken into account. Due to the assessment period in the winter season, only a selected number of season-specific foods were reported in the 4-d FR, whereas the FFQ consists of a fixed food list and the study participants have to estimate their intake under consideration of the respective season. Another limitation of the study was the small sample size, which represents one of the most limiting factors of the current study. A sample size of a minimum of 50 subjects but preferably 100 or more is recommended for validation studies [18]. Sample size post-calculations indicated that with a minimum sample size of 50, the power to detect significant correlations of 0.35, 0.40 and 0.45 would respectively be 0.74, 0.85 and 0.94 (two-tailed and alpha = 0.05).

Further, we did not use biomarkers or other objective reference measures to assess validity, which presents a major limitation of this study. The FFQ assessed dietary intake over a period of four weeks and inclusion of concentration biomarkers in plasma or in adipose tissue would have added valuable information about its validity [38–40]. Nonetheless, there is a lack of biomarkers to reflect wider aspects of dietary intake, and the use of biomarkers for validation of dietary assessment methods is costly.

In conclusion, the 127-food itemed self-administered FFQ showed moderate relative validity for protein and various foods such as fruits, egg, meat, sausage, nuts, salty snacks, beverages such as water, tea and coffee, soft drinks with sugar and alcoholic beverages, thus showing comparable results with other FFQ validation studies and acceptable validity for the other macronutrients and frequently consumed food groups. Therefore, it can be considered as an appropriate tool to assess and characterize usual dietary intake of adults in epidemiological studies. But in these studies, the observed gender differences in under- and over-reporting of specific food items and groups may need to be considered in interpreting observed gender differences in the association between nutrition and health.
